# Von Brun’s Bandage

**Published:** 1886-03

**Authors:** E. J. Beall

**Affiliations:** Fort Worth, Texas


### VON BRUN'S BANDAGE.

Fort Worth, Texas, March 6. 1886.

Ed. Daniel's Texas Medical Journal:

I HAVE received a number ef letters of inquiry as to the mode of preparation of Von Brun's bandage or gauze, mentioned several times in my report as chairman of Section on Surgery at the Houston meeting of Texas State Medical Association.

Will you be kind enough to publish the following:

| | | |
|---|---|---|
| ℞ | Carbolic acid | 10 parts. |
| | Resin | 40 " |
| | Castor oil | 4 " |
| | Alcohol | 200 " |

The resin is to be pounded and slowly added to alcohol, with constant stirring. When solution is complete, add carbolic acid and castor oil. In this menstruum I place cheese cloth. After a few hours, or when needed, tear into widths desirable, roll and keep in oiled paper or rubber tissue. I use this commonly over borated, sublimated, or salicylated cotton, or gauze, or jute, or wood wool, etc.

Now and then I make the bandage by adding large amounts of resin. Then I find the same useful in fractures of forearm of children, having premised starched binders' board, etc.

This can be cheapened by substituting benzine for the alcohol.

E. J. BEALL, M. D.

*Note.*—See page 187-189, Pilcher on Wound Treatment.

